# Frequency of leisure activity engagement and health functioning over a 4-year period: a population-based study amongst middle-aged adults

**DOI:** 10.1186/s12889-022-13670-3

**Published:** 2022-06-30

**Authors:** Esme Elsden, Feifei Bu, Daisy Fancourt, Hei Wan Mak

**Affiliations:** 1 Department of Epidemiology and Public Health, University College London, 1-19 Torrington Place, London, WC1E 7HB England; 2 Department of Behavioural Science and Health, University College London, 1-19 Torrington Place, London, WC1E 7HB England

**Keywords:** Leisure activities, SF-36, 1970s British birth cohort, Health functioning, Frequency of engagement

## Abstract

**Rationale:**

Leisure activities have wide-ranging benefits for physical and mental health. However, previous studies have often focused on “leisure” as a homogeneous group of activities. This study was therefore designed to take a prospective and comparative approach exploring different types of leisure activities, as well as investigating whether frequency of engagement is associated with strength of benefits.

**Method:**

Data from the 1970 British Cohort Study Waves 9 (age 42) and 10 (age 46) were analysed (*N* = 5,639). Eight domains derived from the SF-36 health survey questionnaire were used to measure health functioning (general health, vitality, bodily pain, social functioning, physical functioning, mental health, role limitations due to emotional, and role limitations due to physical problems). Leisure activities included physical activity, culture engagement, arts participation, volunteering or community engagement, and literature activities. Both ordinary least squares and logistic regressions were applied.

**Results:**

Physical activity was associated with greater levels of physical functioning, general health, and vitality at higher frequencies, while cultural engagement was associated with social functioning and physical functioning when engaged in several times a year. Arts participation and literature activities had a general negative association with health functioning. Engagements in volunteering/community groups showed varying associations with health functioning (both positive and negative) depending on the levels of engagements.

**Conclusion:**

This research suggests that the types of leisure activities and levels of engagement can have differential associations with health amongst middle-aged adults. This may be helpful for public health initiatives and programmes such as social prescribing schemes when formulating programmes, especially regarding ‘dosage’ of engagement. Further, the overall benefits of high engagement frequency suggest that increasing leisure engagement could play an important role in supporting improving health and wellbeing at a population level.

**Supplementary Information:**

The online version contains supplementary material available at 10.1186/s12889-022-13670-3.

## Introduction

It has been well-documented that leisure activities have wide-ranging benefits for physical and mental health, and wellbeing [1, 2]. Leisure activities are defined as voluntary, non-work, enjoyable activities engaged in during free time, such as reading for pleasure, physical activity, volunteering, being part of community groups, engaging in creative activities, and going to cultural venues or events [3]. It has been shown that these activities can help improve life satisfaction and mental health functioning [4, 5], reduce stress [6], facilitate self-esteem [7], provide cognitive stimulation [8], promote social engagement [9], reduce sedentary behaviours associated with depression [10] and support coping skills [11–13]. Engagement in leisure activities has also been shown to help prevent the development of health conditions including coronary heart disease, cognitive decline, dementia and chronic pain [14–23]. Furthermore, for individuals experiencing health conditions such as chronic stress and depression, leisure activities have been shown to help manage symptoms [6, 24]. There is also a growing body of evidence suggesting that these activities may help increase longevity [25–30]. Over 600 mechanisms of action underlying these associations between leisure, mental and physical health outcomes have been identified, including biological mechanisms (e.g. increasing brain activation, changing hormone levels, modulating brain biomarkers), psychological mechanisms (e.g. supporting coping, enhancing meaning in life, developing self- and group- identities), behavioural mechanisms (e.g. reducing unhealthy activity engagements, increasing motivation to healthy habits), and social mechanisms (e.g. increasing social contact, building social capital, supporting group cohesion) [1].

However, many of the studies exploring the relationship between leisure and health have focused on leisure activities as a homogenous group. Yet ‘leisure’ is an umbrella term that encompasses a wide range of varied activities. Various classifications have been used to distinguish between different leisure activities, such as ‘relaxed’ leisure (e.g. listening to music or reading) vs ‘serious’ leisure that requires more rigorous pursuit (e.g. sports, arts and hobbies) [31], ‘active’ participation (e.g. volunteering) vs ‘passive’ engagement (e.g. going to cultural venues [32]), and social leisure (e.g. going to community groups) vs solitary leisure (e.g. home-based crafts [33, 34]). The presence or absence of these different components such as commitment, arousal and social engagement likely leads to different health and wellbeing outcomes. For example, studies into cognitive function have shown greater benefits for cognitively demanding and physical leisure engagement than other types [35]. Similarly, studies into stress response have found greater biological benefits from active than passive engagement [36]. Studies of wellbeing have also shown greater benefits for life satisfaction from creative leisure activities compared to other types of leisure [37]. Studies focusing specifically on physical activity have shown benefits for mental health functioning in older adults [38]. These studies highlight that different types of leisure activities could relate to different domains of health outcomes. Yet there remains little research that compares the relationship between different types of leisure and multiple dimensions of health functioning. Consequently, it remains unclear what components of leisure are responsible for such beneficial associations, so there is a clear need for research that takes a comparative approach exploring different types of leisure alongside one another.

Another consideration is that individuals engaging in one activity may also be more likely to engage in other similar types of activities (e.g., arts participation and cultural engagement). For example, one study found that there are recognised patterns of ‘omnivore’ cultural behaviours wherein people already engaging in some leisure activities such as singing and playing a musical instrument are also likely to engage in others such as dramatic arts [39]. Sociological research has also found that people with certain socio-demographics, cultural tastes and preferences and social recognition of status are more likely to engage in specific types of cultural activities [40, 41]. Similarly, social psychology research has shown that people who engage in one social leisure activity such as community group membership are likely to be members of other groups too, and membership of multiple groups appears to be beneficial for health and wellbeing [42]. Failing to consider various types of activities simultaneously might lead to over- or under-estimation of the effect of the activities on health/wellbeing [43]. Therefore, studies are needed that simultaneously model the relationship between multiple different types of leisure and health outcomes to compare their relative effects.

In addition, it remains unclear how frequently one should be engaging in these activities to experience the benefits of them. It has been shown that, for example, people participating in the arts and attending cultural events at the highest frequency experienced the most benefits for improved mental health function and life satisfaction and decreased mental distress [4]. Similarly, it has been found that *regular* physical activity (vs infrequent engagement) is needed to improve health and wellbeing and prevent and manage diseases such as hypertension, diabetes and heart disease [44]. Differentiating people who engage ‘frequently’ from those who ‘rarely’ engage is therefore essential to understand how health and wellbeing benefits may vary across engagement levels. This is particularly relevant for adults in their peak of mid-life (ages between 40 and 50 years) as this is a critical period for poorer population mental health outcomes (including the development of cognitive decline and early dementia) due to reduced leisure time, increased work-related stress, and social and familial changes such as marriage breakdown, empty nests, increasing caring responsibilities, and decreasing physical health due to aging [45]. In addition, as people at this age group are likely to be employed, their leisure engagement levels and the associated health outcomes may be different to other age groups especially when their leisure activities resemble their job role such as skills, activities, and mental and physical demands, diminishing the “recovery” benefits the leisure activities bring. Understanding the frequency of different leisure activities engagement could also help inform policymakers to design and implement more robust public health initiatives to fit the needs of middle-aged adults.

In light of this, the present study examined the association between leisure activity engagement (i.e., physical activity, cultural engagement, arts participation, volunteering and community groups, and literature activities) and health functioning using the 36-Item Short Form Health Survey (SF-36) amongst middle-aged adults. This measure attempts to capture a more holistic understanding of a person’s health via both mental and physical health functioning, and how this impacts their daily life by differentiating between multiple aspects of health including different aspects of functioning, somatic symptoms such as pain and fatigue, and general assessments of health. As with taking a multi-faceted approach to exploring types of leisure, this multi-faceted approach to exploring health outcomes is important to help further understand the mechanisms by which leisure activities affect health and wellbeing. It also has the potential to improve the use of social prescribing schemes that signpost leisure pursuits and activities to patients by identifying appropriate types of activities and engagement levels that could benefit specific health outcome domains.

## Data & method

This study used data from the 1970 British Cohort Study (BCS70), which is a prospective longitudinal cohort study that follows a representative sample of people born in England, Scotland and Wales in a single week of 1970 across their life course (*N* = 17,198) [46]. Data collected from this study include biomarkers, health data, physical characteristics, educational and social development as well as economic circumstances and socioeconomic position. Since the birth survey in 1970, there have been multiple surveys (or waves/sweeps) conducted including at ages 5, 10, 16, 26, 30, 34, 38, 42 and 46. BCS70 has ethical approval from the NHS Multi-Centre Research Ethics Committee (MREC) with all participants giving fully informed consent.

This study used data from BCS70 Wave 9 at age 42 (2012) and Wave 10 at age 46 (2016). There were 9,841 participants at Wave 9 (response rate 74.6%) and 8,581 participants at Wave 10 (response rate 70%). 7,924 participants completed both waves, and amongst them, 6,280 responded to the exposure (i.e., leisure activities) and the outcome (i.e., SF-36) variables. In our analysis, we only considered those who provided valid responses to all other covariate measures, which gives us a total analytical sample of 5,639 participants.

### Measures

#### Leisure activities

Self-reported leisure questionnaire was measured in Wave 9 (age 42). The questionnaire consisted of 34 items. Informed by prior studies, we grouped these items into 5 broad leisure activities [2] and for each group of activities, the highest frequency of engagement recorded for each category was used as a measure of a participant’s engagement in that domain: (i) *physical activity* included health, fitness, gym or conditioning activities; swimming or diving; cycling, BMX or mountain biking; dancing; jogging, cross-country, road-running; rambling/walking for pleasure; racquet sports; team sports; marital arts, boxing, wrestling; water sports; horse riding; yoga/pilates; golf; ski-ing; and other sporting activity (collapsed into a 5-point scale: never/less often, 2 to 3 times a month, 1 to 3 days a week, 4 to 5 days a week, everyday); (ii) *culture engagement* included going to a museum; art exhibition/gallery; heritage site/stately home; cinema; theatre to watch a play/drama; theatre to watch a pantomime or musical; opera, classical music concert or ballet; and another type of concert (collapsed into a 4-point scale: never/less often, at least once a year, several times a year, at least once a month/week); (iii) *arts participation* included playing a musical instrument; performing arts; painting, drawing, printmaking or sculpture; photography, film or video making as an artistic activity; and textile crafts, wood crafts or any other crafts (collapsed into a 5-point scale: never, less often than/at least once a year, several times a year, at least once a month, at least once a week); (iv) *volunteering/community groups* included doing unpaid voluntary work; and attending meetings for local groups/voluntary organisations (collapsed into a 5-point scale: never, less often, at least once a year/several times a year, at least once a month/at least once a week); and (v) *literature activities* combined going to a library; book club; reading books in spare time; and writing stories, plays or poetry (collapsed into a 5-point scale: never or less often, at least once a year, several times a year, at least once a month, at least once a week) (Table 1). All leisure activities, except for those grouped in the ‘physical activity’ category, largely involve low intensity of physical activity.

**Table 1:** Leisure activity variables grouping (*N* = 5, 639)

Leisure activity variables	Leisure activities included	Frequency	%
**Physical activity**	Health, fitness, gym or conditioning activities; swimming or diving; cycling, BMX or mountain biking; dancing; jogging, cross-country, road-running; rambling/walking for pleasure; racquet sports; team sports; marital arts, boxing, wrestling; water sports; horse riding; yoga/pilates; golf; ski-ing; other sporting activity	Never/less often	20.3%
		2 to 3 times a month	15.5%
		1 to 3 days a week	42.5%
		4 to 5 days a week	11.5%
		Every day	10.3%
**Culture engagement**	Going to a museum; art exhibition/gallery; heritage site/stately home; cinema; theatre to watch a play/drama; theatre to watch a pantomime or musical; opera, classical music concert or ballet; another type of concert	Never/less often	7.47%
		At least once a year	19.0%
		Several times a year	61.9%
		At least once a month/week	11.7%
**Arts participation**	Playing a musical instrument; performing arts; painting, drawing, printmaking or sculpture; photography, film or video making as an artistic activity; textile crafts, wood crafts or any other crafts	Never	43.6%
		Less often than/ at least once a year	16.7%
		Several times a year	16.7%
		At least once a month	10.1%
		At least once a week	14.0%
**Volunteering/ community groups**	Doing unpaid voluntary work; attending meetings for local groups/voluntary organisations	Never	52.6%
		Less often	13.3%
		At least once a year/several times a year	17.9%
		At least once a month/at least once a week	16.1%
**Literature activities**	Reading books in spare time; going to a library; going to a book club; writing stories, plays or poetry	Never or less often	12.0%
		At least once a year	9.70%
		Several times a year	15.8%
		At least once a month	20.3%
		At least once a week	42.2%

#### Mental and physical health functioning

Mental and physical health functioning was measured using SF-36 (Ware et al. 1993) in Wave 10 (age 46). The SF-36 survey is comprised of 36 items which are grouped into nine individual domains. These include (i) physical functioning (PF), which assesses how limited in performing daily activities including bathing and dressing due to health problems; (ii) social functioning (SF), which assesses whether individuals have frequent interference with normal social activities due to physical or emotional problems; (iii) bodily pain (BP), whether individuals have very severe and extremely limiting pain; (iv) general health (GH), which evaluates personal health as poor and believes it is likely to get worse; (v) vitality (VT) that assesses whether an individual feels tired or worn out all the time; (vi) mental health (MH) domain measures feelings of nervousness or depression all the time; (vii) role limitations due to emotional (RE) or (viii) physical problems (RP) that assess whether an individual has reduced functioning in their daily activities because of emotional or physical problems; and (ix) health transition (HT), which measures whether participants report any changes in their general health compared to a previous time point [47].

Except for HT (comparisons between two time-points were not feasible due to data unavailability in Wave 9), the BCS70 derived the remaining eight domains from the question items included in the questionnaire, following the manual instructions set by Ware et al. (1993). Recent methodological studies suggest that the eight domains should not be collapsed into one scale since they independently measure various aspects of health conditions [48]. Instead, we used each domain individually, with each domain scored from 0 to 100, with higher scores indicating better functioning in that domain (e.g., greater social functioning, lower bodily pain, greater vitality) [48]. The only two exceptions were RP and RE, for which the scales were highly skewed and hence were coded as binary variables, indicating if participants had no limiting illness/condition or disability (1 = fully role functioning with a score of 100, 0 = not fully role functioning with a score of < 100) [47]. Full details on the distribution of each scale are presented in Supplementary Fig. 1.

#### Covariates

In the analysis, we adjusted for a set of demographic backgrounds, socio-economic characteristics and health status covariates measured in Wave 9 (age 42), which might confound the association between leisure activities and health functioning. Demographics included gender (woman vs man), ethnicity (ethnic minorities vs white), partnership status (married/civil partnership vs not married/civil partnership), whether participants were living alone, and whether participants had children (including those living in the household). Socio-economic characteristics included occupational status (managerial administrative and professional occupations, intermediate occupations, routine and manual occupations, no economic activity/students/other; the categories were based on the National Statistics Socio-economic 3-category classification (Office for National Statistics, 2020)) [49], education level (no qualification, up to GCSE/O levels/trade apprenticeships or equivalent, up to A level/higher education or equivalent, degree or above), employment status (employed, unemployed, retired/sick/caring or other) and housing tenure (owned vs rented/other). Health status included whether participants had a limiting longstanding mental or physical illness, such as hypertension, backache and migraines, their self-reported health (excellent, very good, good, fair, and poor) and baseline mental health problems (measured using the Malaise Inventory Score) at age 42 [50].

#### Analysis

Different forms of regression analysis were applied depending on the type of outcome variable. Ordinary least squares (OLS) regressions were used to estimate the associations of the frequency of leisure activity engagement with six health functioning domains: GH, VT, BP, SF, PF, and MH. Coefficients and 95% confidence intervals (CIs) were provided to indicate the direction of the relationships. Logistic regressions were used to estimate the associations of the frequency of leisure activity engagement with the remaining two health functioning outcomes: RP and RE. Odds ratios (OR) and 95% CIs were presented to predict how likely participants were to experience fully role functioning based on their leisure activity engagement levels. All models were adjusted for the various leisure activities, demographic backgrounds, socio-economic characteristics, and health status simultaneously. The mean value of VIF is 1.76 across models, suggesting that the risk of collinearity is minimal.

To check the robustness of our results, three sensitivity analyses were carried out: (1) all analyses were repeated while omitting the health status covariates (i.e., self-reported health, malaise inventory score and whether they had a limiting longstanding mental or physical illness) to check how much our health covariates could explain the variations in the outcomes given that SF-36 was not measured in previous waves (Supplementary Table 1); (2) we additionally created an alternative specification for the leisure activity measure and created 5 indexes for each activity by summing the frequency of engagement (Supplementary Table 2); and (3) all analyses were replicated while restricting respondents to those who did not have a limiting long-standing illness (Supplementary Table 3). All analyses were carried out using Stata Version 16 [51].

## Results

In our sample, 54.6% were women and 97.4% were of white ethnic backgrounds. A large proportion of the sample (45.9%) were in the managerial, administrative, and professional occupational group, and 29.5% had a degree or above education qualification. 95.5% of the sample were employed and nearly 79.7% owning their own homes. More than a quarter (27.1%) of participants reported having limiting longstanding mental or physical illness (Table 2).

**Table 2:** Descriptive statistics of analytical sample

	**(*N* = 5,639)** **% /mean (SD)**
*Demographic backgrounds*	
Gender	
Man	45.4%
Woman	54.6%
Ethnicity	
White	97.4%
Ethnic minorities	2.62%
Partnership Status	
Not Married/civil partnership	33.9%
Married/civil partnership	66.1%
Do they live alone?	
Yes	20.4%
No	79.6%
Do they have children?	
Yes	72.7%
No	27.3%
*Socio-economic characteristics*	
Socioeconomic status (NS-SEC)	
Managerial, administrative, and professional occupations	45.9%
Intermediate occupations	20.4%
Routine and manual occupations	21.4%
No economic activity/students/other	12.3%
Education level	
No qualification	18.5%
Up to GCSE/O level or equivalent	36.0%
Up to A level/higher education or equivalent	16.0%
Degree or above	29.5%
Employment status	
Employed	95.5%
Unemployed	1.72%
Retired/sick/caring/education/training/other	2.80%
Tenure	
Owned	79.7%
Rented/other	20.3%
*Health status*	
Limiting longstanding mental or physical illness	
Yes	27.1%
No	72.9%
Self-reported health	
Excellent	23.7%
Very Good	38.7%
Good	25.0%
Fair	9.26%
Poor	3.28%
Mental health problems (Malaise Inventory Score)	1.76 (1.91)
*Outcome measures*	
SF-36 (All continuous domains range from 0–100)	
Physical Functioning Score (PF)	88.3 (20.7)
Social Functioning Score (SF)	86.7 (22.7)
Bodily Pain Score (BP)	79.0 (23.3)
General Health Score (GH)	68.3 (21.5)
Energy/Fatigue Score (VT)	57.7 (21.8)
Emotional Wellbeing Score (MH)	75.1 (18.9)
Role limitations due to physical health (RP)	
Yes	22.3%
No	77.7%
Role limitations due to emotional problems (RE)	
Yes	23.4%
No	76.6%

### Physical activity

Frequent physical activity was positively associated with PF, GH, and VT four years later. For all, there were significant associations for engagement 1–3 days a week and a dose response relationship with more frequent engagement associated with higher functioning. But coefficients were slightly lower for daily engagement for VT. There was no evidence that physical activity was associated with SF, BP, MH, RE or RP (Table 3).

**Table 3:** Regressions estimating the association between frequency of leisure activity engagement at age 42 and the eight domains of SF-36 measured at age 46 (*N* = 5,639)

Leisure activity variables	SF-36 domains							
	PF	SF	BP	GH	VT	MH	RE	RP
Physical activity	Coef (95%CI)	Coef (95%CI)	Coef (95%CI)	Coef (95%CI)	Coef (95%CI)	Coef (95%CI)	OR (95%CI)	OR (95%CI)
2 to 3 times a month	1.10 (-0.50-2.72)	-0.12 (-1.80-1.56)	1.17 (-0.62-2.96)	0.65 (-0.80-2.11)	0.92 (-0.71-2.55)	0.12 (-1.27-1.51)	1.11 (0.88-1.39)	0.96 (0.76-1.21)
1 to 3 days a week	2.31** (0.99-3.63)	-0.09 (-1.47-1.28)	1.05 (-0.41-2.52)	2.89*** (1.70-4.09)	2.47*** (1.13-3.81)	0.15 (-0.98-1.29)	0.98 (0.81-1.18)	0.96 (0.79-1.17)
4 to 5 days a week	2.56** (0.78-4.34)	-0.98 (-2.84-0.88)	1.15 (-0.84 – 3.13)	3.90*** (2.28-5.51)	4.48*** (2.67-6.28)	-0.62 (-2.15-0.92)	1.00 (0.77-1.29)	0.94 (0.72-1.22)
Everyday	2.80** (0.97-4.63)	-0.34 (-2.26-1.57)	1.85 (-0.19-3.89)	4.52*** (2.86-6.18)	3.93*** (2.07 – 5.80)	0.77 (-0.81-2.35)	1.23 (0.94-1.61)	1.07 (0.81-1.40)
Culture engagement								
At least once a year	0.17 (-1.89-2.24)	1.60 (-0.56-3.76)	-0.29 (-2.02-2.59)	1.36 (-0.51-3.24)	0.34 (-1.76-2.44)	0.30 (-1.49 – 2.09)	0.80 (0.59-1.08)	1.08 (0.80-1.45)
Several times a year	2.33* (0.41-4.24)	3.23** (1.23-5.23)	0.62 (-1.51-2.76)	1.73 (-0.00-3.47)	0.37 (-1.58-2.31)	1.36 (-0.29 – 3.01)	0.83 (0.63-1.10)	1.13 (0.85-1.48)
At least once a month/at least once a week	1.12 (-1.19-3.43)	1.41 (-1.00-3.82)	1.33 (-1.25-3.90)	1.86 (-0.23-3.94)	1.06 (-1.28-3.40)	0.77 (-1.22-2.76)	0.91 (0.65-1.28)	1.29 (0.92-1.81)
Arts participation								
Less often/at least once a year	-0.39 (-1.78 – 0.99)	-0.14 (-1.59-1.30)	-1.28 (-2.83 – 0.26)	-1.01 (-2.26-0.25)	0.00 (-1.41-1.41)	-0.10 (-1.30-1.09)	0.89 (0.73-1.09)	0.82 (0.67 – 1.01)
Several times a year	0.42 (-0.96-1.81)	-1.31 (-2.76-0.13)	-1.72* (-3.26--0.18)	-1.61* (-2.87--0.36)	-0.12 (-1.52-1.29)	-0.28 (-1.47-0.92)	0.90 (0.74-1.10)	0.77* (0.63-0.95)
At least once a month	-1.70* (-3.39--0.01)	-1.54 (-3.31-0.23)	-2.48* (-4.37--0.60)	-2.21** (-3.74--0.68)	-0.15 (-1.87-1.57)	0.41 (-1.05-1.87)	0.81 (0.64 – 1.03)	0.79 (0.62 – 1.02)
At least once a week	-2.16** (-3.67--0.65)	-2.07* (-3.65--0.49)	-3.00*** (-4.69--1.32)	-3.26*** (-4.63--1.89)	-1.04 (-2.57-0.50)	-0.64 (-1.94-0.67)	0.77* (0.62-0.95)	0.69** (0.56-0.86)
Volunteering/community groups								
Less often	-0.16 (-1.63-1.31)	-1.14 (-2.67-0.40)	-0.92 (-2.56-0.72)	0.11 (-1.23-1.44)	-0.03 (-1.52-1.47)	-0.48 (-1.75-0.79)	0.80* (0.65-0.98)	0.80* (0.65-0.99)
At least once a year/several times a year	-0.30 (-1.63 – 1.04)	-1.51* (-2.90--0.12)	0.53 (-0.95 – 2.01)	-0.05 (-1.26-1.15)	0.57 (-0.78-1.92)	-0.16 (-1.31 – 0.99)	0.73** (0.61-0.88)	0.98 (0.80-1.19)
At least once a month/at least once a week	-0.09 (-1.48-1.29)	0.06 (-1.38-1.51)	-0.17 (-1.72-1.37)	0.63 (-0.63-1.88)	1.77* (0.37-3.18)	0.81 (-0.38 – 2.00)	0.89 (0.73-1.09)	0.79* (0.65-0.97)
Literature activities								
At least once a year	1.07 (-0.98-3.12)	-1.60 (-3.74 – 0.55)	-0.87 (-3.16-1.41)	-1.15 (-3.00 – 0.71)	-3.31** (-5.39--1.23)	-2.16* (-3.93 – 0.39)	1.09 (0.80-1.48)	0.82 (0.60 – 1.13)
Several times a year	0.95 (-0.91 – 2.81)	-0.54 (-2.48 – 1.40)	-0.35 (-2.42-1.72)	-2.32** (-4.01--0.64)	-2.66** (-4.55--0.77)	-0.97 (-2.57-0.63)	0.90 (0.68-1.18)	0.70* (0.53 – 0.93)
At least once a month	0.75 (-1.05 – 2.55)	-2.07* (-3.95--0.19)	0.11 (-1.90 – 2.12)	-0.54 (-2.17-1.09)	-2.03* (-3.86--0.20)	-2.83*** (-4.38 – 1.27)	0.78 (0.60-1.01)	0.78 (0.59-1.03)
At least once a week	1.49 (-0.20-3.18)	-0.75 (-2.52-1.01)	-0.26 (-2.14-1.63)	-0.64 (-2.17 – 0.89)	-1.91* (-3.63--0.19)	-2.01** (-3.47 – 0.55)	0.94 (0.73-1.21)	0.76* (0.59 – 0.99)

The reference group for all models is “never”. *** *p* < 0.001, ** *p* < 0.01, * *p* < 0.05. The models were adjusted for gender, ethnicity, partnership status, whether they lived alone, whether they had children, socioeconomic status, education level, employment status, tenure, whether they have a limiting long-standing illness, baseline mental health problems and self-reported health while controlling for the other leisure activities. Outcomes: physical functioning (PF), social functioning (SF), bodily pain (BP; reverse coded), general health (GH), vitality (VT), mental health (MH), role limitations due to physical health problems (RP; coded to 1 = fully role functioning and 0 = not fully role functioning), role limitations due to emotional problems (RE; coded to 1 = fully functioning and 0 = not fully functioning)

### Culture engagement

Culture engagement was positively associated with PF and SF when engaging several times a year. There was no evidence that culture engagement was associated with BP, GH, VT, MH, RE or RP (Table 3).

### Arts participation

Arts participation was found to be negatively associated with PF, SF, BP (reversely coded) and GH for people engaging with arts activities as often as at least once a week. Further, participating in the arts several times a year or as least once a week was also associated with lower odds of fully role functioning (either due to physical or emotional problems). No associations were found for VT and MH (Table 3).

### Volunteering/community groups

Engaging in volunteering and community groups several times a year or less (vs never) was associated with lower levels of SF. However, regular engagement (either weekly/monthly) was positively related to VT. Volunteering/community groups engagement was also associated with lower odds of experiencing full functioning (either due to physical or emotional problems). No associations were found for PF, BP, GH, and MH (Table 3).

### Literature activities

Engaging in literature activities monthly and yearly was associated with lower levels of SF and GH, respectively. Such engagement was also negatively related to VT and MH for almost all frequency (although the relationships became weaker at higher frequencies), and was correlated with lower odds of experiencing full functioning due to physical problems. No associations were found for PF, BP, and RE (Table 3).

### Sensitivity analysis

When repeating the analysis without controlling for health status covariates, some of the associations (particularly with physical activity and cultural engagement) became more pronounced. For instance, physical activity was positively associated with all health functioning, with engagement as infrequent as 2 to 3 times a month was related to higher levels of PH, BP (reversely coded), GH and VT. Similarly, for cultural engagement, it was positively associated with all health outcomes, except for RE. In particular, the associations with SF and GH were apparent with engagement as minimal as at least once a year. Results largely remained for other activities including arts participation, volunteering/community groups and literature activities (Supplementary Table 1).

When converting the leisure activities measures to summed scores for each activity, results largely remained with some small changes. For instance, frequent physical activity were additionally found to be associated with higher levels of MH, and frequent cultural engagement was also shown to be positively associated with BP (reversely coded) and experiencing full role functioning-physical. In contrast, frequent arts participation was additionally found to be related to lower levels of VT. For volunteering/community groups and literature activities, most of the associations were attenuated yet the relationship between literature activities frequency and mental health continued to be seen (Supplementary Table 2).

Finally, when focusing only on respondents without any limiting long-standing illness, results were very similar. This provides stronger evidence for the uni-directional relationship between leisure activities and health functioning outcomes, at least for healthy adults (Supplementary Table 3).

## Discussion

This study examined the associations between leisure engagement and health functioning 4 years later amongst middle-aged adults. We found that frequent physical activity and cultural engagement were associated with improved physical health functioning. Higher levels of physical activity were also strongly associated with general health and vitality whereas cultural engagement was associated with better social functioning. However, some other activities were shown to be associated with poorer health functioning. This included arts participation, where yearly, monthly or weekly engagement was related to poorer physical and social functioning, bodily pain, and poorer general health. Lower odds of fully role functioning (either due to physical or emotional problems) were also found for people who engaged several times a year or weekly. Volunteering/community group engagement was associated with higher vitality for those who engaged monthly or weekly, however those who engaged were also more likely to experience poorer social functioning and role limitations due to emotional/physical problems. Finally, there were some associations at lower frequencies of literature activities engagement with poorer vitality and monthly engagement with poorer mental health.

In line with previous studies [25, 52, 53], physical activity and cultural engagement were associated with better health functioning. Such benefits were found even when the engagement levels were infrequent: 1 to 3 days a week for physical activity and several times a year for cultural engagement. However, these benefits appeared to be most prominent when engaged with routinely, such as every day for physical activity. It is of interest that whilst a dose–response relationship was found for physical activity for both physical functioning and general health, there was a tapering of the relationship for vitality. This could indicate that engaging daily in physical activity caused it to become more tiring; more of a routine than a pleasurable leisure activity [54]. Other potential reasons are that the time commitment involved in daily physical activity may have displaced time for other leisure activities that could have provided greater benefits for vitality [54]. Our study design allowed for the comparison of the features of these leisure activities to help identify the underlying mechanisms related to frequency of engagement of such associations. If we compare physical activity and cultural engagement, similarities and differences in the components of these different leisure activities become apparent. For example, on one hand, both of these activities involve a reduction in sedentary behaviours through outdoors engagement [44, 54] and promote social interactions, which improve health functioning [14, 55]. On the other hand, the fact that physical activity additionally improved general health and vitality whilst cultural engagement had benefits for social functioning suggests that the differences between the activities activated different mechanisms of action connecting them with health outcomes [2]. For instance, cultural engagement can support social functioning through reducing loneliness, whereas physical activity helps specifically improve people’s energy through reducing inflammation, improving muscle strength, and better sleep quality [56, 57]. However, it should be noted that even though we looked at more specific categories of leisure activities than in previous studies, each category nonetheless comprised a wide array of activities ranging from yoga to sports team activity so more work is needed to understand which components of specific activities are associated with particular health outcomes. Also, the contexts (e.g. environment and atmosphere) where these activities take place may also affect the components relating to the outcomes [58]. Further, it is also possible that some of the associations were driven by reverse causality, despite our attempts to control for baseline effects. Yet our sensitivity analyses did suggest that health conditions (including limiting long-standing illness, baseline mental health problems and self-reported health) explained a substantial variation of the association between leisure activities and health functioning. Also, when restricting our sample to those without any limiting long-standing illness, most of the associations remained.

Some of our other findings aligned less well with previous literature. For example, our findings that engagements in arts activities such as playing a musical instrument and painting/drawing weekly were associated with poorer physical and social functioning and poorer general health, bodily pain and role limitations due to physical/emotional problems are in opposition to previous studies showing that these activities have protective mental and physical health effects [59, 60]. In considering why this might be, in addition to reverse causality aforementioned, two explanations might help in the interpretation of the findings. First, people who engaged with arts activities during mid-life might be using the arts to cope with their daily stress caused by roles and responsibilities (e.g., parenting, employment, partnership, a change in their health) [45, 61]. Future studies are needed to investigate whether the effects of arts participation found in our study were influenced by ‘age’, ‘life stages’ or ‘cohort’ effect. Second, participants with relatively high levels of engagement might themselves be working in the arts and cultural sector (e.g., musicians or arts practitioners) and might experience job-related stress or fatigue. Indeed, it has indeed been shown in previous studies that professional artists and musicians tend to have poorer health functioning [62]. Literature activities also showed similar patterns. Those who engaged in these activities tended to have lower levels of social functioning, general health, vitality and mental health. They were also more likely to experience role limitations due to physical health problems. Yet, it is important to note that benefits of engagement in literature activities may vary due to the wide-ranging activities. For instance, it may be plausible that ‘active’ and ‘social’ activities such as becoming a member of a book club may yield greater benefits due to greater exposure to social interactions than ‘passive’ and ‘solitary’ activities such as those who write poems or stories. It has also been proposed that ‘serious’ leisure, through which people attain specific skills and have particular goals, may have greater effects on people’s health [63]. But these theories remain to be tested in future studies.

Finally, we found some inconsistency in volunteering/community groups. People who engaged less in volunteering/community groups tended to experience poorer social functioning and role limitations due to physical/emotional health problems, whereas those who engaged monthly/weekly were more likely to have higher levels of vitality (although also more likely to experience role limitations due to physical problems). The inconsistency suggests that solely focusing on engagement itself (i.e. whether or not engaged) may not suffice if we were to use these activities to improve health functioning, yet the levels of engagement (i.e. how much of engagement) may be key. If low levels of engagement were associated with poorer functioning (as suggested in our study), boosting engagement through creating more varied opportunities to engage and providing opportunities that can be woven into routine may help improve people’s wellbeing in their mid-life. Further, benefits of engagement in volunteering/community groups may vary depending on the types and purposes of the activities. For instance, volunteering locally in charity shops versus participating in national voluntary social action activities such as climate change and social justice are likely to have different impacts on people’s health and wellbeing.

The study has a number of strengths, including using data from a nationally representative British birth cohort study. Furthermore, the comprehensive measures on leisure activities (both by type and frequency) and health functioning (using the SF-36) allow us to detail the ways different activities related to the varying domains of health functioning. The use of the SF-36 allows us to further previous work on mechanisms and the specificity of frequency and type of engagement that provide a more comprehensive view of health outcomes. However, this study is not without limitations. First, whilst our study analysed data from two time points, the direction of the association cannot be established. We were unable to adjust for the SF-36 measure at baseline as data were not available in previous waves. Nonetheless, our models controlled for whether or not people had a limiting longstanding mental or physical illness at baseline as well as baseline mental health status and self-reported health, alongside with a sensitivity analysis restricting to those without a limiting long-standing illness, which hopefully could help estimate the associations between leisure activities and SF-36 more accurately. Further, due to data constraints, we were not able to examine whether the changes in health functioning were associated with the changes in the engagement levels of leisure activities, which could be explored in future research. Given that the length and types of participation in leisure activities might vary according to age and life stages, the association between such participation and health or wellbeing may change accordingly. Future research is thus needed to identify whether the findings presented here are found at other life stages. In addition, more studies are required to compare and contrast group- and individual- based leisure activities and how they associate with health functioning. Finally, it is important to note that while the rich and high-quality data have enabled various statistical tests to examine the relationships more comprehensively, spurious associations might have been existed. Further studies with more applied statistical techniques are encouraged to detect such associations.

## Conclusion

In conclusion, there is emerging evidence that different leisure activities support physical and mental health in different ways and through different mechanisms. Physical activity and culture engagement showed the strongest positive associations with the SF-36 health domains. Physical exercise was associated with better physical functioning, general health and vitality, especially when engagement was frequent, while cultural engagement was associated with social functioning and physical functioning when engaged with several times a year. However, arts participation, volunteering/community group engagement, and literature activities had varying effects on health functioning depending on the levels of engagement. Further research is warranted, especially when the age 50 wave of the 1970’s British Cohort study is released, and comparisons can be made with previous waves. Policy implications of this research are also grounded in helping to direct public health initiatives such as social prescribing schemes, which already have a focus on using leisure activities to support health and wellbeing amongst middle-aged individuals. Our research provides social prescribing practitioners with empirical evidence of how various leisure activities might help support different health conditions and provides more detail on the ‘dosage’ of engagement needed. Given that our study shows that higher frequency is generally associated with stronger and better health functioning, identifying factors that can support engagement and barriers to engagement that can be removed could be crucial to improve wellbeing amongst middle-aged adults at a population level.

## Supplementary Information

**Additional file 1:** **Supplementary Table 1.** Sensitivity analysis omitting health status variables from the models (*N*=5,715). **Supplementary Table 2.** Sensitivity analysis using the frequency index of leisure activity engagement (*N*=4,475). **Supplementary Table 3.** Sensitivity analysis restricting to respondents without any limiting long-standing illness (*N*=4,113). **Supplementary Fig. 1.** The distribution of each eight domain of SF-36. 

## Data Availability

The 1970 British Cohort Study data set is publicly available via the UK Data Service: https://beta.ukdataservice.ac.uk/datacatalogue/series/series?id=200001
