# Supplementary material for: Frequency of leisure activity engagement and health functioning over a 4-year period: a population-based study amongst middle-aged adults
Source: BMC Public Health. 2022 Jun 30;22:1275. doi: 10.1186/s12889-022-13670-3 (PMC9248182; doi:10.1186/s12889-022-13670-3)

| **Supplementary Table 1: Sensitivity analysis omitting health status variables from the models (N=5,715)** | | | | | | | | |
| --- | --- | --- | --- | --- | --- | --- | --- | --- |
| Leisure activity variables | SF-36 domains | | | | | | | |
|  | PF | SF | BP | GH | VT | MH | RE | RP |
| Physical activity | Coef (95%CI) | Coef (95%CI) | Coef (95%CI) | Coef (95%CI) | Coef (95%CI) | Coef (95%CI) | OR (95%CI) | OR (95%CI) |
| 2 to 3 times a month | 2.70** | 1.60 | 2.74** | 2.69** | 2.33* | 1.15 | 1.23 | 1.13 |
|  | (0.98 - 4.41) | (-0.29 - 3.49) | (0.79 - 4.69) | (0.89 - 4.49) | (0.46 - 4.20) | (-0.47 - 2.77) | (1.00 - 1.52) | (0.91 - 1.39) |
| 1 to 3 days a week | 4.91*** | 3.15*** | 4.04*** | 7.09*** | 5.68*** | 2.52*** | 1.23* | 1.29** |
|  | (3.51 - 6.30) | (1.61 - 4.69) | (2.45 - 5.62) | (5.63 - 8.55) | (4.16 - 7.20) | (1.20 - 3.84) | (1.04 - 1.46) | (1.08 - 1.53) |
| 4 to 5 days a week | 5.69*** | 2.91** | 4.94*** | 9.45*** | 8.89*** | 2.71** | 1.34* | 1.36* |
|  | (3.79 - 7.58) | (0.83 – 5.00) | (2.80 – 7.09) | (7.47 - 11.4) | (6.83 - 10.9) | (0.92 - 4.49) | (1.06 - 1.69) | (1.07 - 1.74) |
| Everyday | 5.22*** | 2.46* | 4.49*** | 8.57*** | 7.05*** | 2.95** | 1.43** | 1.33* |
|  | (3.27 - 7.17) | (0.32 - 4.61) | (2.28 - 6.70) | (6.54 - 10.6) | (4.93 - 9.17) | (1.11 - 4.78) | (1.12 - 1.83) | (1.04 - 1.71) |
| Culture engagement |  |  |  |  |  |  |  |  |
| At least once a year | 1.58 | 3.44** | 1.55 | 3.21** | 2.10 | 1.93 | 0.96 | 1.21 |
|  | (-0.63 - 3.80) | (1.00 - 5.88) | (-0.96 – 4.07) | (0.89 - 5.52) | (-0.31 - 4.51) | (-0.16 – 4.02) | (0.73 - 1.25) | (0.93 - 1.59) |
| Several times a year | 4.02*** | 5.85*** | 2.55* | 4.45*** | 3.19** | 3.88*** | 1.07 | 1.33* |
|  | (1.97 – 6.06) | (3.60 – 8.10) | (0.23 - 4.88) | (2.30 - 6.59) | (0.96 - 5.41) | (1.95 - 5.82) | (0.83 - 1.38) | (1.04 - 1.71) |
| At least once a month/At least once a week | 2.38 | 3.69** | 2.95* | 3.90** | 3.45* | 2.81* | 1.09 | 1.39* |
|  | (-0.09 - 4.84) | (0.97 - 6.40) | (0.15 - 5.75) | (1.32 - 6.48) | (0.77 – 6.13) | (0.49 – 5.14) | (0.80 - 1.47) | (1.03 - 1.89) |
| Arts participation |  |  |  |  |  |  |  |  |
| Less often/at least once a year | -0.84 | -1.06 | -2.17* | -2.28** | -0.82 | -0.72 | 0.86 | 0.77** |
|  | (-2.32 - 0.65) | (-2.69 - 0.57) | (-3.86 - -0.49) | (-3.83 - -0.73) | (-2.43 - 0.80) | (-2.12 - 0.68) | (0.71 - 1.03) | (0.64 - 0.93) |
| Several times a year | 0.20 | -1.65* | -2.19* | -2.06** | -0.25 | -0.28 | 0.90 | 0.77** |
|  | (-1.28 - 1.68) | (-3.28 - -0.02) | (-3.87 - -0.51) | (-3.61 - -0.52) | (-1.85 - 1.36) | (-1.68 - 1.12) | (0.75 - 1.09) | (0.64 - 0.93) |
| At least once a month | -1.99* | -2.28* | -3.06** | -2.90** | -0.43 | 0.15 | 0.80* | 0.78* |
|  | (-3.81 - -0.18) | (-4.28 - -0.28) | (-5.12 - -1.01) | (-4.79 - -1.00) | (-2.40 - 1.54) | (-1.55 - 1.86) | (0.64 - 1.00) | (0.62 - 0.98) |
| At least once a week | -3.04*** | -3.60*** | -4.37*** | -4.93*** | -1.94* | -1.35 | 0.75** | 0.64*** |
|  | (-4.66 - -1.43) | (-5.38 - -1.82) | (-6.21 - -2.54) | (-6.62 - -3.24) | (-3.70 - -0.18) | (-2.87 - 0.18) | (0.61 - 0.91) | (0.53 - 0.79) |
| Volunteering/community groups | | | | | | | | |
| Less often | -0.69 | -1.63 | -1.55 | -0.84 | -0.67 | -0.90 | 0.79* | 0.77** |
|  | (-2.27 - 0.89) | (-3.37 - 0.11) | (-3.34 - 0.25) | (-2.49 - 0.82) | (-2.39 - 1.05) | (-2.39 - 0.59) | (0.65 - 0.95) | (0.63 - 0.94) |
| At least once a year/several times a year | -0.10 | -0.94 | 0.89 | 0.35 | 0.91 | 0.13 | 0.79** | 1.01 |
|  | (-1.53 - 1.32) | (-2.51 - 0.63) | (-0.73 - 2.51) | (-1.14 - 1.84) | (-0.64 - 2.46) | (-1.21 - 1.47) | (0.66 - 0.94) | (0.84 - 1.21) |
| At least once a month/At least once a week | -0.21 | 0.17 | -0.07 | 0.63 | 2.01* | 1.09 | 0.91 | 0.80* |
|  | (-1.68 - 1.27) | (-1.46 - 1.79) | (-1.74 - 1.60) | (-0.91 – 2.18) | (0.41 - 3.62) | (-0.30 - 2.48) | (0.75 - 1.09) | (0.67 - 0.97) |
| Literature activities |  |  |  |  |  |  |  |  |
| At least once a year | 0.60 | -1.65 | -0.93 | -1.34 | -3.24** | -2.07 | 1.05 | 0.83 |
|  | (-1.58 – 2.79) | (-4.06 – 0.76) | (-3.41 - 1.55) | (-3.63 – 0.95) | (-5.62 - -0.87) | (-4.13 – -0.00) | (0.79 - 1.39) | (0.62 - 1.11) |
| Several times a year | 1.30 | 0.20 | 0.30 | -1.34 | -1.98 | -0.44 | 0.92 | 0.78 |
|  | (-0.68 – 3.29) | (-1.98 – 2.39) | (-1.95 - 2.56) | (-3.42 – 0.74) | (-4.13 - 0.18) | (-2.32 - 1.43) | (0.72 - 1.19) | (0.60 - 1.02) |
| At least once a month | 0.77 | -1.40 | 0.37 | -0.01 | -1.18 | -1.94* | 0.84 | 0.83 |
|  | (-1.16 – 2.69) | (-3.52 – 0.72) | (-1.81 – 2.56) | (-2.03 - 2.00) | (-3.27 – 0.92) | (-3.75 – 0.12) | (0.66 - 1.07) | (0.65 - 1.08) |
| At least once a week | 1.80 | -0.03 | 0.31 | 0.51 | -0.87 | -1.13 | 1.00 | 0.83 |
|  | (-0.01 – 3.60) | (-2.02 – 1.96) | (-1.74 - 2.36) | (-1.38 – 2.40) | (-2.83 - 1.09) | (-2.83 – 0.58) | (0.79 - 1.26) | (0.66 - 1.06) |
| Note: The reference group for all models is “never”. *** p<0.001, ** p<0.01, * p<0.05. The models were adjusted for gender, ethnicity, partnership status, whether they lived alone, whether they had children, socioeconomic status, education level, employment status, and tenure while controlling for the other leisure activities. Outcomes: physical functioning (PF), social functioning (SF), bodily pain (BP; reverse coded), general health (GH), vitality (VT), mental health (MH), role limitations due to physical health problems (RP; coded to 1=fully role functioning and 0=not fully role functioning), role limitations due to emotional problems (RE; coded to 1=fully role functioning and 0=not fully role functioning). | | | | | | | | |

| **Supplementary Table 2: Sensitivity analysis using the frequency index of leisure activity engagement (N=4,475)** | | | | | | | | |
| --- | --- | --- | --- | --- | --- | --- | --- | --- |
| Leisure activity variables (index) | SF-36 domains | | | | | | | |
|  | PF | SF | BP | GH | VT | MH | RE | RP |
|  | Coef (95%CI) | Coef (95%CI) | Coef (95%CI) | Coef (95%CI) | Coef (95%CI) | Coef (95%CI) | OR (95%CI) | OR (95%CI) |
| Physical activity | 0.27*** | 0.02 | -0.00 | 0.34*** | 0.41*** | 0.10* | 1.00 | 0.99 |
|  | (0.15 - 0.38) | (-0.11 - 0.14) | (-0.13 - 0.13) | (0.24 - 0.45) | (0.29 - 0.52) | (0.00 - 0.21) | (0.98 - 1.01) | (0.98 - 1.01) |
| Culture engagement | 0.13* | 0.15* | 0.14* | 0.08 | 0.08 | 0.10 | 0.99 | 1.02* |
|  | (0.02 - 0.24) | (0.03 - 0.27) | (0.01 - 0.27) | (-0.02 - 0.19) | (-0.04 - 0.20) | (-0.00 - 0.20) | (0.97 - 1.01) | (1.00 - 1.04) |
| Arts participation | -0.22** | -0.25** | -0.30*** | -0.37*** | -0.18* | -0.12 | 0.98* | 0.96*** |
|  | (-0.36 - -0.09) | (-0.40 - -0.10) | (-0.46 - -0.14) | (-0.50 - -0.24) | (-0.33 - -0.04) | (-0.24 - 0.00) | (0.96 - 0.99) | (0.94 - 0.98) |
| Volunteering/community groups | -0.18 | -0.11 | -0.02 | -0.05 | 0.15 | 0.07 | 0.99 | 0.98 |
|  | (-0.37 - 0.02) | (-0.32 - 0.10) | (-0.24 - 0.21) | (-0.23 - 0.13) | (-0.06 - 0.35) | (-0.10 - 0.25) | (0.96 - 1.02) | (0.95 - 1.01) |
| Literature activities | 0.25 | -0.02 | 0.08 | -0.02 | -0.10 | -0.25* | 0.99 | 0.98 |
|  | (-0.00 - 0.50) | (-0.30 - 0.25) | (-0.21 - 0.37) | (-0.25 - 0.21) | (-0.36 - 0.17) | (-0.48 - -0.03) | (0.95 - 1.02) | (0.94 - 1.01) |
| Note: The reference group for all models is “never”. *** p<0.001, ** p<0.01, * p<0.05. The models were adjusted for gender, ethnicity, partnership status, whether they lived alone, whether they had children, socioeconomic status, education level, employment status, tenure, whether they have a limiting long-standing illness, baseline mental health problems and self-reported health while controlling for the other leisure activities. Outcomes: physical functioning (PF), social functioning (SF), bodily pain (BP; reverse coded), general health (GH), vitality (VT), mental health (MH), role limitations due to physical health problems (RP; coded to 1=fully role functioning and 0=not fully role functioning), role limitations due to emotional problems (RE; coded to 1=fully role functioning and 0=not fully role functioning). Leisure activity exposure variables were indexes of frequency. | | | | | | | | |

| **Supplementary Table 3: Sensitivity analysis restricting to respondents without any limiting long-standing illness (N=4,113)** | | | | | | | | |
| --- | --- | --- | --- | --- | --- | --- | --- | --- |
| Leisure activity variables | SF-36 domains | | | | | | | |
|  | PF | SF | BP | GH | VT | MH | RE | RP |
| Physical activity | Coef (95%CI) | Coef (95%CI) | Coef (95%CI) | Coef (95%CI) | Coef (95%CI) | Coef (95%CI) | OR (95%CI) | OR (95%CI) |
| 2 to 3 times a month | 0.39 | -0.49 | -0.04 | 0.93 | 0.33 | -0.33 | 1.09 | 1.09 |
|  | (-1.33 – 2.10) | (-2.21 – 1.24) | (-1.95 – 1.88) | (-0.71 – 2.57) | (-1.54 – 2.20) | (-1.89 – 1.23) | (0.82 – 1.45) | (0.80 – 1.47) |
| 1 to 3 days a week | 1.97** | -0.89 | 0.26 | 2.85*** | 1.83* | -0.47 | 0.90 | 0.90 |
|  | (0.56 – 3.37) | (-2.30 – 0.53) | (-1.31 – 1.83) | (1.51 – 4.19) | (0.29 – 3.36) | (-1.74 – 0.81) | (0.71 – 1.14) | (0.71 – 1.15) |
| 4 to 5 days a week | 2.48* | -1.04 | 1.45 | 4.46*** | 4.23*** | -1.04 | 0.92 | 1.00 |
|  | (0.59 – 4.37) | (-2.93 – 0.86) | (-0.66 – 3.56) | (2.65 – 6.26) | (2.16 – 6.30) | (-2.76 – 0.68) | (0.67 – 1.26) | (0.71 – 1.40) |
| Everyday | 1.75 | -0.52 | 1.41 | 4.14*** | 3.53** | 0.20 | 1.02 | 1.04 |
|  | (-0.21 – 3.72) | (-2.49 – 1.46) | (-0.79 – 3.61) | (2.27 – 6.02) | (1.38 – 5.68) | (-1.59 – 1.99) | (0.73 – 1.42) | (0.73 – 1.49) |
| Culture engagement |  |  |  |  |  |  |  |  |
| At least once a year | -0.83 | 0.84 | -0.64 | 0.30 | 0.06 | -0.02 | 0.70 | 0.98 |
|  | (-3.08 – 1.41) | (-1.42 – 3.10) | (-3.16 – 1.87) | (-1.84 – 2.45) | (-2.40 – 2.52) | (-2.06 – 2.03) | (0.47 – 1.03) | (0.67 – 1.44) |
| Several times a year | 1.55 | 2.27* | 0.64 | 1.44 | 0.24 | 1.31 | 0.73 | 1.07 |
|  | (-0.54 – 3.64) | (0.16 – 4.37) | (-1.70 – 2.98) | (-0.56 – 3.43) | (-2.05 – 2.53) | (-0.60 – 3.21) | (0.50 – 1.07) | (0.75 – 1.53) |
| At least once a month/at least once a week | 0.57 | 1.04 | 2.50 | 1.35 | 1.37 | 0.96 | 0.78 | 1.25 |
|  | (-1.95 – 3.09) | (-1.50 – 3.58) | (-0.33 – 5.32) | (-1.07 – 3.76) | (-1.40 – 4.13) | (-1.34 – 3.26) | (0.50 – 1.21) | (0.81 – 1.95) |
| Arts participation |  |  |  |  |  |  |  |  |
| Less often/at least once a year | -0.69 | -0.86 | -1.39 | -0.80 | -0.54 | -0.82 | 0.83 | 0.74* |
|  | (-2.14 – 0.75) | (-2.31 – 0.60) | (-3.01 – 0.23) | (-2.18 – 0.58) | (-2.13 – 1.04) | (-2.14 – 0.50) | (0.66 – 1.06) | (0.57 – 0.95) |
| Several times a year | 0.13 | -1.80* | -1.38 | -1.76* | -0.34 | -0.83 | 0.87 | 0.79 |
|  | (-1.34 – 1.59) | (-3.28 - -0.33) | (-3.03 – 0.26) | (-3.16 - -0.36) | (-1.94 - 1.26) | (-2.17 – 0.50) | (0.68 – 1.11) | (0.61 – 1.02) |
| At least once a month | -1.63 | -1.16 | -1.49 | -1.46 | 0.76 | 0.87 | 0.79 | 0.85 |
|  | (-3.42 – 0.17) | (-2.97 – 0.64) | (-3.50 – 0.52) | (-3.18 – 0.25) | (-1.20 – 2.73) | (-0.76 – 2.51) | (0.59 – 1.06) | (0.62 – 1.18) |
| At least once a week | -2.43** | -2.60** | -2.50** | -2.89*** | -1.77 | -1.53* | 0.68** | 0.63** |
|  | (-4.06 - -0.79) | (-4.25 - -0.95) | (-4.34 - -0.67) | (-4.46 - -1.33) | (-3.56 – 0.02) | (-3.02 - -0.04) | (0.52 – 0.88) | (0.47 – 0.83) |
| Volunteering/community groups | | | | | | | | |
| Less often | -0.30 | -0.58 | -1.22 | -0.20 | 0.32 | -0.25 | 0.85 | 0.77 |
|  | (-1.87 – 1.26) | (-2.15 – 1.00) | (-2.97 – 0.54) | (-1.70 – 1.30) | (-1.40 – 2.03) | (-1.67 – 1.18) | (0.66 – 1.09) | (0.59 – 1.01) |
| At least once a year/several times a year | 0.00 | -0.72 | 0.54 | -0.57 | 0.69 | 0.31 | 0.83 | 0.97 |
|  | (-1.40 – 1.41) | (-2.14 – 0.69) | (-1.04 – 2.12) | (-1.91 – 0.78) | (-0.86 – 2.23) | (-0.98 – 1.59) | (0.66 – 1.05) | (0.75 – 1.24) |
| At least once a month/at least once a week | 0.66 | 0.54 | 0.29 | 0.77 | 1.64* | 0.99 | 1.09 | 0.86 |
|  | (-0.82 – 2.13) | (-0.95 – 2.02) | (-1.36 – 1.95) | (-0.64 - 2.18) | (0.02 – 3.26) | (-0.35 – 2.34) | (0.85 – 1.41) | (0.66 – 1.11) |
| Literature activities |  |  |  |  |  |  |  |  |
| At least once a year | 1.28 | -1.25 | -1.44 | -1.08 | -3.20** | -1.33 | 1.13 | 0.78 |
|  | (-0.86 - 3.42) | (-3.41 – 0.90) | (-3.84 – 0.95) | (-3.12 – 0.97) | (-5.54 - -0.86) | (-3.28 – 0.62) | (0.77 – 1.64) | (0.52 – 1.15) |
| Several times a year | 0.57 | -0.41 | -1.54 | -2.81** | -2.94** | -0.80 | 0.87 | 0.66* |
|  | (-1.37 – 2.52) | (-2.37 – 1.55) | (-3.73 – 0.64) | (-4.67 - -0.94) | (-5.07 - -0.80) | (-2.58 – 0.97) | (0.63 – 1.22) | (0.46 – 0.95) |
| At least once a month | 1.31 | -1.93* | -0.57 | -1.12 | -1.95 | -2.20* | 0.78 | 0.67* |
|  | (-0.59 – 3.21) | (-3.84 - -0.16) | (-2.69 – 1.56) | (-2.94 – 0.69) | (-4.04 - 0.13) | (-3.93 - -0.47) | (0.57 – 1.08) | (0.47 – 0.94) |
| At least once a week | 1.36 | -0.79 | -1.73 | -1.63 | -1.91 | -1.34 | 1.00 | 0.71* |
|  | (-0.43 – 3.16) | (-2.59 – 1.02) | (-3.74 – 0.28) | (-3.34 - 0.09) | (-3.88 – 0.05) | (-2.97 – 0.29) | (0.73 – 1.36) | (0.51 – 0.99) |
| Note: The reference group for all models is “never”. *** p<0.001, ** p<0.01, * p<0.05. The models were adjusted for gender, ethnicity, partnership status, whether they lived alone, whether they had children, socioeconomic status, education level, employment status, tenure, baseline mental health problems and self-reported health while controlling for the other leisure activities. Outcomes: physical functioning (PF), social functioning (SF), bodily pain (BP; reverse coded), general health (GH), vitality (VT), mental health (MH), role limitations due to physical health problems (RP; coded to 1=fully role functioning and 0=not fully role functioning), role limitations due to emotional problems (RE; coded to 1=fully role functioning and 0=not fully role functioning). | | | | | | | | |

**Supplementary Fig. 1: The distribution of each eight domain of SF-36.**


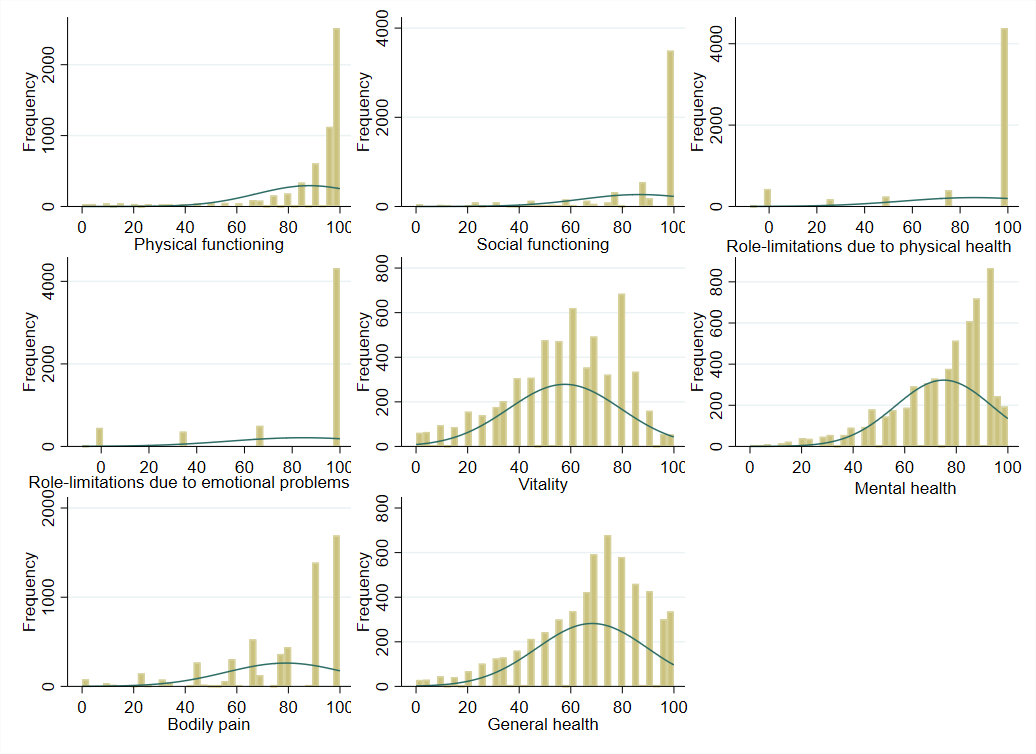

Supplement: Supplementary file 1 — Additional file 1: Supplementary Table 1. Sensitivity analysis omitting health status variables from the models (N=5,715). Supplementary Table 2. Sensitivity analysis using the frequency index of leisure activity engagement (N=4,475). Supplementary Table 3. Sensitivity analysis restricting to respondents without any limiting long-standing illness (N=4,113). Supplementary Fig. 1. The distribution of each eight domain of SF-36. [file 12889_2022_13670_MOESM1_ESM.docx]
